# Retraction: Effects of “mindfulness acceptance insight commitment” training on flow state and mental health of college swimmers: a randomized controlled experimental study

**DOI:** 10.3389/fpsyg.2024.1369142

**Published:** 2024-01-18

**Authors:** Frontiers Editorial Office

Following publication, concerns were raised regarding possible data fabrication in the article. An investigation was conducted in accordance with Frontiers' policies. The authors failed to provide a satisfactory explanation for the lack of raw data and admitted to generating data to create their figures. As a result, the conclusions of the article have been deemed unreliable and the article is retracted.

The retraction was approved by the Specialty Chief Editor of Movement Science and the Chief Executive Editor of Frontiers. The authors disagree with the retraction.

